# Intensity-modulated radiotherapy provides better quality of life than two-dimensional conventional radiotherapy for patients with stage II nasopharyngeal carcinoma

**DOI:** 10.18632/oncotarget.17582

**Published:** 2017-05-03

**Authors:** Xin-Bin Pan, Shi-Ting Huang, Kai-Hua Chen, Yan-Ming Jiang, Jia-Lin Ma, Song Qu, Ling Li, Long Chen, Xiao-Dong Zhu

**Affiliations:** ^1^ Department of Radiation Oncology, Cancer Hospital of Guangxi Medical University, Nanning, Guangxi 530021, P.R. China

**Keywords:** nasopharyngeal carcinoma, quality of life, intensity-modulated radiotherapy, two-dimensional conventional radiotherapy

## Abstract

Two-dimensional conventional radiotherapy (2D-CRT) and intensity-modulated radiotherapy (IMRT) are effective for control of nasopharyngeal carcinoma (NPC). The purpose of this study was to compare the quality of life (QoL) of stage II NPC patients treated with 2D-CRT versus IMRT. We conducted a cross-sectional study of 106 patients with stage II NPC treated with 2D-CRT (n = 47) versus IMRT (n = 59) between June 2008 and June 2013. For all subjects, disease-free survival was more than 3 years. QoL was assessed using the European Organization for Research and Treatment of Cancer Quality of Life Questionnaire-Core 30 (EORTC QLQ-C30) questions and the Head and Neck 35 (EORTC QLQ-H&N35) questions. Patients receiving IMRT with or without concurrent chemotherapy had better outcomes in head and neck related symptoms and general aspects of QoL than those receiving 2D-CRT with or without concurrent chemotherapy. Thus, IMRT improves the QoL of patients with stage II NPC as compared to 2D-CRT.

## INTRODUCTION

Nasopharyngeal carcinoma (NPC) is highly endemic in southern China [1]. Radiotherapy is the primary treatment modality for NPC. Two-dimensional conventional radiotherapy (2D-CRT) has been effective in controlling NPC, but complications to organs at risk resulting from 2D-CRT are severe and lifelong. In the last decade, intensity-modulated radiotherapy (IMRT) has rapidly replaced 2D-CRT due to its technical and dosimetric superiority, and when resources permit, has become the most commonly used radiation technique for NPC.

The incidence of stage II NPC has greatly increased with improvements in diagnosis, and after treatment the 5-year overall survival is assumed to be 95% or higher [2, 3]. The high survival rate makes quality of life (QoL) increasingly important. Previous studies suggested that IMRT improved symptoms and QoL for NPC survivors [4–6]. However, all of these studies were confounded by the interference of chemotherapy [7, 8]. Moreover, while the previous studies treated NPC as a whole group and analyzed QoL, most patients had advanced loco-regional NPC. Only one randomized controlled trial compared QoL of IMRT versus 2D-CRT in early stage NPC [9]. This trial suggested that IMRT was superior with regard to speech problems and swallowing. However, another randomized controlled trial reported that there was no significant difference in patient-reported xerostomia between IMRT and 2D-CRT [10]. Moreover, in both studies the sample size was relatively small, and the follow-up time was only 1 year, and neither provided accurate information regarding the QoL of IMRT versus 2D-CRT in early stage NPC.

In developing regions, many patients are treated with 2D-CRT rather than IMRT because they have no access to IMRT or the financial burden of IMRT is too great. Although 2D-CRT provides a similar survival benefit for NPC as IMRT [11, 12], clinicians have begun to pay more attention to QoL. We conducted a cross-sectional study to compare the QoL between IMRT and 2D-CRT in patients with stage II NPC. The result of this study might help clinicians make treatment decisions and provide information to health workers on which health services are most beneficial.

## RESULTS

### Patient characteristics

Of 106 stage II NPC patients, 47 received 2D-CRT and 59 received IMRT. Disease-free survival of all subjects was more than 3 years. Table 1 summarizes patients’ characteristics.

**Table 1:** Patient characteristics

	2D-CRT (n=47)	IMRT (n=59)
Gender		
Male	31(65.96%)	39(66.10%)
Female	16(34.04%)	20(33.90%)
Age (years)		
Median	44	42
Range	25-68	22-64
Follow-up (months)		
Median	64	50
Range	44-89	38-61
AJCC stage		
T1N1M0	11(23.40%)	10(16.95%)
T2N0M0	15(31.91%)	9(15.25%)
T2N1M0	21(44.69%)	40(67.80%)
Chemotherapy		
Yes	14(29.79%)	37(62.71%)
No	33(70.21%)	22(37.29%)

2D-CRT: two-dimensional conventional radiotherapy; IMRT: intensity-modulated radiotherapy.

### QoL of IMRT versus 2D-CRT for the whole group

In the whole group, IMRT (n=59) had higher mean scores in head and neck related symptoms and broad aspects of QoL for patients with stage II NPC than 2D-CRT (n=47) (Table 2). Clinical superiority of IMRT for QoL was significant on all functional scales and most symptom scales upon clinical interpretation (difference in mean scores≥10 points).

**Table 2:** Mean quality of life scores of 2D-CRT versus IMRT for the whole group

Scales	2D-CRT (n=47)		IMRT (n=59)		t	p
	Mean	SD	Mean	SD		
EORTC QLQ-C30						
Global quality of life	65.07	16.08	81.21	15.59	−4.939	0.000
Physical functioning	75.74	19.33	92.2	13.43	−4.792	0.000
Role functioning	74.47	22.21	90.43	14.64	−4.112	0.000
Emotional functioning	67.38	26.74	89.01	15.65	−4.786	0.000
Cognitive functioning	60.99	29.34	87.59	13.22	−5.666	0.000
Social functioning	61.35	24.35	91.13	13.39	−7.348	0.000
Fatigue	34.28	22.2	12.53	17.89	5.23	0.000
Nausea/emesis	4.26	8.84	1.06	4.12	2.244	0.028
Pain	18.09	17.32	8.16	11.97	3.233	0.002
Dyspnea	11.35	15.97	4.96	16.99	1.877	0.064
Insomnia	38.3	25.99	19.15	24.81	3.654	0.000
Appetite loss	15.6	18.19	1.42	9.72	4.714	0.000
Constipation	7.09	18.31	3.55	17.35	0.964	0.338
Diarrhea	9.22	17.99	2.84	9.4	2.155	0.035
Financial problems	44.68	27.17	21.28	24.5	4.386	0.000
EORTC QLQ-H&N35						
Pain	11.52	12.35	3.37	5.4	4.147	0.000
Swallowing	25.71	17.62	5.32	8.93	7.076	0.000
Senses	25.18	17.68	11.7	14.29	4.064	0.000
Speech	10.64	11.57	2.84	7.13	3.934	0.000
Social contact	28.9	22.51	4.79	10.95	6.603	0.000
Social eating	12.77	12.1	2.55	6.6	5.078	0.000
Sexuality	54.96	32.02	20.92	20.7	6.121	0.000
Teeth	44.68	29.71	13.48	19.24	6.044	0.000
Opening mouth	29.08	23.69	7.09	13.79	5.499	0.000
Dry mouth	58.16	22.49	22.7	25.16	7.204	0.000
Sticky saliva	9.93	18.28	4.26	16.47	1.581	0.117
Coughing	13.48	17.94	12.06	17.62	0.387	0.7
Feeling ill	22.7	20.97	8.51	17.68	3.545	0.001
Pain killers	2.84	9.4	2.84	9.4	0.000	1.000
Nutritional supplements	23.4	18.28	12.06	16.19	3.186	0.002
Feeding tube	0.000	0.000	0.000	0.000	0.000	1.000
Weight loss	5.67	12.66	1.42	6.8	2.03	0.046
Weight gain	2.13	8.24	7.8	14.27	−2.361	0.021

2D-CRT: two-dimensional conventional radiotherapy; IMRT: intensity-modulated radiotherapy; SD: standard deviation; EORTC QOL-C30: European Organization for Research and Treatment of Cancer Quality of Life Questionnaire-Core 30; EORTC QOL-H&N35: The EOTRC Quality of Life Questionnaire-Head and Neck 35.

### QoL of IMRT versus 2D-CRT without concurrent chemotherapy

In the radiotherapy alone subgroup, IMRT (n=22) had better QoL outcomes than 2D-CRT (n=33), except on scales of nausea/emesis, diarrhea, sticky saliva, coughing, pain killers, feeding tube, weight loss, and weight gain. Differences of most scales between the two groups were significant (Table 3).

**Table 3:** Mean quality of life scores of 2D-CRT versus IMRT without concurrent chemotherapy

Scales	2D-CRT (n=33)		IMRT (n=22)		t	p
	Mean	SD	Mean	SD		
EORTC QLQ-C30						
Global quality of life	69.95	15.30	86.74	11.69	−4.364	0.000
Physical functioning	80.61	19.10	97.58	8.11	−4.528	0.000
Role functioning	80.81	20.46	98.48	4.90	−4.761	0.000
Emotional functioning	74.49	23.75	95.08	14.47	−3.990	0.000
Cognitive functioning	66.67	30.33	94.70	9.47	−4.959	0.000
Social functioning	66.67	24.65	96.97	6.58	−6.712	0.000
Fatigue	26.94	19.84	6.06	8.21	5.391	0.000
Nausea/emesis	4.04	9.35	1.52	4.90	1.306	0.197
Pain	13.64	17.90	5.30	7.95	2.350	0.023
Dyspnea	9.09	15.08	1.52	7.11	2.500	0.016
Insomnia	28.28	20.62	12.12	21.93	2.777	0.008
Appetite loss	14.14	18.69	0.00	0.00	4.346	0.000
Constipation	8.08	20.46	0.00	0.00	2.268	0.030
Diarrhea	7.07	18.18	1.52	7.11	1.584	0.120
Financial problems	39.39	28.20	9.09	15.19	5.152	0.000
EORTC QLQ-H&N35						
Pain	10.86	14.05	1.52	4.18	3.590	0.001
Swallowing	22.22	18.00	1.89	5.10	6.129	0.000
Senses	23.74	17.69	6.06	8.21	4.990	0.000
Speech	9.43	11.82	1.52	3.90	3.564	0.001
Social contact	24.24	22.57	.38	1.78	6.046	0.000
Social eating	12.12	11.72	.30	1.42	5.730	0.000
Sexuality	47.47	30.08	11.36	20.82	5.260	0.000
Teeth	40.40	32.01	9.09	18.35	4.599	0.000
Opening mouth	27.27	25.62	1.52	7.11	5.468	0.000
Dry mouth	54.55	23.30	16.67	19.92	6.249	0.000
Sticky saliva	7.07	16.15	1.52	7.11	1.739	0.089
Coughing	10.10	17.65	10.61	18.93	−0.101	0.920
Feeling ill	18.18	20.57	6.06	16.70	2.400	0.020
Pain killers	3.03	9.73	0.00	0.00	1.789	0.083
Nutritional supplements	21.21	20.10	6.06	13.16	3.378	0.001
Feeding tube	0.00	0.00	0.00	0.00	0.000	1.000
Weight loss	2.02	8.08	1.52	7.11	0.238	0.813
Weight gain	1.01	5.80	0.00	0.00	0.814	0.419

2D-CRT: two-dimensional conventional radiotherapy; IMRT: intensity-modulated radiotherapy; SD: standard deviation; EORTC QOL-C30: European Organization for Research and Treatment of Cancer Quality of Life Questionnaire-Core 30; EORTC QOL-H&N35: The EOTRC Quality of Life Questionnaire-Head and Neck 35.

### QoL of IMRT versus 2D-CRT with concurrent chemotherapy

In the concurrent chemotherapy subgroup, IMRT (n=37) had better QoL outcomes than 2D-CRT (n=14), except for symptoms of nausea/emesis, dyspnea, constipation, sticky saliva, pain killers, and feeding tube. Differences of most scales between the two groups were significant (Table 4).

**Table 4:** Mean quality of life scores of 2D-CRT versus IMRT with concurrent chemotherapy

Scales	2D-CRT (n=14)		IMRT (n=37)		t	p
	Mean	SD	Mean	SD		
EORTC QLQ-C30						
Global quality of life	53.57	11.65	73.2	15.48	−4.294	0.000
Physical functioning	64.29	14.93	86.31	13.96	−4.933	0.000
Role functioning	59.52	19.3	83.33	15.21	−4.628	0.000
Emotional functioning	50.6	26.65	79.95	18.37	−4.48	0.000
Cognitive functioning	47.62	22.51	77.48	15.82	−5.334	0.000
Social functioning	48.81	19.02	82.43	16.17	−6.313	0.000
Fatigue	51.59	17.76	20.12	19.92	5.178	0.000
Nausea/emesis	4.76	7.81	1.35	6.06	1.474	0.157
Pain	28.57	10.19	10.36	13.24	4.643	0.000
Dyspnea	16.67	17.3	6.31	18.98	1.78	0.081
Insomnia	61.9	22.1	24.32	23.11	5.243	0.000
Appetite loss	19.05	17.12	2.7	12.12	3.276	0.004
Constipation	4.76	12.1	4.5	19.5	0.046	0.964
Diarrhea	14.29	17.12	2.7	9.22	2.403	0.029
Financial problems	57.14	20.37	35.14	27.16	2.747	0.008
EORTC QLQ-H&N35						
Pain	13.1	7.1	6.31	6.92	3.106	0.003
Swallowing	33.93	14.05	11.26	10.25	6.349	0.000
Senses	28.57	17.82	13.06	15.28	3.09	0.003
Speech	13.49	10.83	3	7.7	3.319	0.004
Social contact	39.88	18.83	11.94	13.26	5.096	0.000
Social eating	14.29	13.3	3.78	7.46	2.794	0.013
Sexuality	72.62	30.39	32.43	21.14	5.35	0.000
Teeth	54.76	21.11	23.42	22.03	4.583	0.000
Opening mouth	33.33	18.49	15.32	21.65	2.959	0.006
Dry mouth	66.67	18.49	28.83	25.05	5.135	0.000
Sticky saliva	16.67	21.68	4.5	17.85	1.872	0.076
Coughing	21.43	16.57	9.91	15.45	2.33	0.024
Feeling ill	33.33	18.49	9.01	16.94	4.464	0.000
Pain killers	2.38	8.91	3.6	10.49	−0.386	0.701
Nutritional supplements	28.57	12.1	16.22	16.89	2.898	0.007
Feeding tube	0.000	0.000	0.000	0.000	0.000	1
Weight loss	14.29	17.12	0.9	5.48	2.87	0.012
Weight gain	4.76	12.1	14.41	16.74	−2.273	0.03

2D-CRT: two-dimensional conventional radiotherapy; IMRT: intensity-modulated radiotherapy; SD: standard deviation; EORTC QOL-C30: European Organization for Research and Treatment of Cancer Quality of Life Questionnaire-Core 30; EORTC QOL-H&N35: The EOTRC Quality of Life Questionnaire-Head and Neck 35.

## DISCUSSION

This study suggests that IMRT has better outcomes in both functional and symptom scales of EORTC QLQ-C30 compared to 2D-CRT with or without concurrent chemotherapy. The result indicates that IMRT should be provided to NPC patients, irrespective of a concomitant substantial increase in expenditures if resources permit.

We observed that 2D-CRT adversely affected patients with regard to symptom scales, global QoL, and functional scales compared to IMRT for the whole group. Differences of most functional and symptom scales were significant upon clinical interpretation. The result was similar to previous studies [4–6]. However, patients included in the previous studies were mostly T3-4 or N2-3. Radiotherapy combined with chemotherapy is the primary treatment modality for advanced loco-regionally NPC. It was suggested that concurrent chemotherapy adversely affected QoL of NPC patients [7]. None of these studies could totally exclude the interference of chemotherapy on QoL. In order to exclude the interference of chemotherapy, we conducted a subgroup analysis to compare the QoL of IMRT versus 2D-CRT without concurrent chemotherapy. The result revealed that IMRT alone significantly improved the QoL compared to 2D-CRT alone. Moreover, our subgroup result also suggested that IMRT had better QoL than 2D-CRT with concurrent chemotherapy.

It has been suggested that IMRT has significantly lower radiation-induced toxicity than 2D-CRT [13], but the change in the patient-reported xerostomia scores or QoL may be not statistically different between the two groups [10]. Possibilities to explain this inconsistency are as follow: (1) QoL assessment may contain questions that are not specific to RT-induced toxicities. (2) The criteria used to differentiate between grade 1 and grade 2 of QoL is rather vague and subjective. (3) Physician and patient bias may exist in an unblinded randomization setting. (4) Previous studies used a small sample size and a relatively shorter follow-up time. However, this study shows that IMRT has better QoL with or without concurrent chemotherapy in a longer follow-up time. The result further confirms that lower radiation-induced toxicities of IMRT may produce better QoL compared to 2D-CRT [9].

IMRT increases the expenses for NPC treatment and eventually increases the financial difficulties of individuals in developing countries such as China. Some studies found that financial difficulties adversely affected QoL [14, 15]. Consequently, IMRT would adversely affect QoL. However, we found that patients receiving 2D-CRT suffered from greater financial difficulties than those receiving IMRT. The potential interpretation was that patients received 2D-CRT because of financial difficulties. Financial burden after treatment gave patients receiving 2D-CRT worse QoL, but the relationship between financial problems and QoL is still unclear. Further controlled studies should be performed to test the interference of financial difficulties on QoL.

The result of our study should be interpreted with caution. The EORTC QLQ-H&N35 may have some limitations in assessment of QoL of NPC patients. NPC has different biological characteristics and treatment than other head and neck cancers. Xerostomia, deafness, otitis media, and symptoms from organs at risk injury after radiotherapy are the main symptoms in NPC survivors. Although the EORTC QLQ-H&N35 is a specific questionnaire assessing the QoL of head and neck cancer, it does not deal with adverse radiation effects well enough.

Limitations of this study should be considered: (1) The small sample size (106 patients) may lead to statistical error. (2) This study assessed the QoL at only one time point. A more methodologically sound approach would employ a longitudinal design in which the same individuals are assessed repeatedly at various time points.

In conclusion, this study suggests that IMRT improves most general aspects of QoL for patients with stage II NPC compared to 2D-RCT. IMRT is a better treatment technique for stage II NPC.

## MATERIALS AND METHODS

### Patients

We analyzed QoL data of patients with stage II NPC in the Cancer Hospital of Guangxi Medical University from June 2008 to June 2013. Inclusion criteria were (1) pathologically proven NPC, (2) stage II NPC per the 7th Edition of the UICC/AJCC staging system, (3) receiving radical radiotherapy or concurrent chemotherapy, and (4) disease-free survival >3 years. Exclusion criteria were (1) age >70 or <18 years, (2) recurrent or metastatic NPC, (3) receiving induced or adjuvant chemotherapy, (4) a second malignancy, except for cured skin basal cell carcinoma or early stage cervical cancer, (5) severe cerebral, cardiac, hematologic, renal, hepatic, or mental disease, or (6) an incomplete self-reporting questionnaire.

From June 2008 to June 2013, 235 patients with stage II NPC received radical treatment at the Cancer Hospital of Guangxi Medical University. There were 129 total excluded patients; 8 were lost to follow-up, 4 received induced chemotherapy, 40 received adjuvant chemotherapy, 5 died, 9 were loco-regional failures, 7 were distant failures, 51 were non-compliant, and 5 did not complete the questionnaire. This study finally included 106 patients treated with IMRT (n = 59) or 2D-CRT (n = 47).

### Radiotherapy

Patients received 2D-CRT in two phases. In the first phase, patients were irradiated by 6-megavolt bilateral and opposing photon beams. The dose for the faciocervical field and lower anterior cervical field was 36 Gy. In the second phase, the dose for primary tumor was boosted from 66 Gy to 70 Gy. The prescribed irradiation dose was 2 Gy per fraction with 5 daily fractions per week.

Patients received IMRT per the International Commission on Radiation Units and Measurements Report 62 guidelines. Gross tumor volume (GTVnx) and cervical lymph node tumor volume (GTVnd) were determined by CT/MRI. Clinical target volume (CTV) included the GTV with a 1-cm to 1.5-cm margin, the entire nasopharyngeal space, and the positive lymph node regions. The prescribed radiation dose was 66 Gy to 70.06 Gy in 30 to 31 fractions for GTV, and 54 Gy to 60 Gy in 30 fractions for CTV with 5 daily fractions per week.

### Chemotherapy

Patients received concurrent chemotherapy on days 1, 22, and 43 during radiotherapy. The chemotherapy regimen was cisplatin 100 mg/m^2^/d by intravenous infusion. Chemotherapy was postponed or discontinued for patients who experienced serious toxicity and could not recover before the next schedule.

### QoL measurement

Patients’ QoL data were obtained by two clinicians from our department, both of whom received a uniform training. A subset of the patients was instructed to answer the questions during the visit to our clinic. Most patients were assessed by telephone. QoL data of all patients was analyzed by a third investigator. Consent was obtained from all patients included in the study.

QoL assessment used the Chinese version of the European Organization for Research and Treatment of Cancer Quality of Life Questionnaire-Core 30-questions (EORTC QLQ-C30) and the Head and Neck 35-questions (EORTC QLQ-H&N35) [16–19]. The EORTC QLQ-C30 is a cancer-specific questionnaire containing a global QoL score, five functional scales, three symptom scales, and six single items. The QLQ-H&N35 is a site-specific questionnaire assessing QoL of head-and-neck cancer patients. The QLQ-H&N35 contains seven multiple-item and six single-item scales. The standard score of all scales ranges from 0 to 100. A high score for a global QoL or functional scale represents a high/healthy level of global QoL or functioning, whereas a high score for a symptom scale represents a symptom problem. QoL changes of ≥10 points were considered clinically relevant [20, 21].

### Statistical analysis

Statistical analysis was performed using SPSS for Windows version 16.0 (SPSS Inc., Chicago, IL). The T-test was used to compare the mean scores of QoL between two groups. All significance tests were two-sided and P value <0.05 was considered statistically significant.

### Ethics statement

This cross-sectional study was approved by the Ethics Committee of Cancer Hospital of Guangxi Medical University.

## Abbreviations

NPC: nasopharyngeal carcinoma
2D-CRT: two-dimensional conventional radiotherapy
IMRT: intensity-modulated radiotherapy
QoL: quality of life
GTV: gross tumor volume
CTV: clinical target volume
EORTC QOL-C30: European Organization for Research and Treatment of Cancer; Quality of Life Questionnaire-Core 30
EORTC QOL-H&N35: The EOTRC Quality of Life Questionnaire-Head and Neck 35.
